# Central auditory abilities are associated with cognitive and structural neuroimaging markers of Alzheimer’s disease dementia

**DOI:** 10.1002/alz.089753

**Published:** 2025-01-03

**Authors:** Meher Lad, Timothy Griffiths, John‐Paul Taylor

**Affiliations:** ^1^ Newcastle University, Newcastle upon Tyne, Tyne and Wear United Kingdom; ^2^ Newcastle University, Newcastle‐upon‐Tyne, Tyne and Wear United Kingdom; ^3^ Newcastle University, Newcastle upon Tyne United Kingdom

## Abstract

**Background:**

Hearing loss is associated with cognitive and neuroimaging markers of Alzheimer’s disease dementia but it is unclear how specific measures relate to these after accounting for a range of hearing abilities.

**Method:**

**200 participants (155 cognitively normal, 25 mild cognitively impaired and 20 Alzheimer’s disease dementia) underwent auditory testing (peripheral and central abilities), cognitive testing and MR scanning (structural and diffusion‐weighted sequences) to evaluate the relationship between hearing, cognition and imaging brain measures**.

**Result:**

**Central auditory measures such as speech‐in‐noise perception and auditory memory for longer durations were associated with cognitive impairment across the Alzheimer’s disease continuum and specific auditory measures were independently associated with morphometric and diffusion‐weighted brain measures**.

**Conclusion:**

**Auditory cognition could serve as a unique marker of cognition in Alzheimer’s disease dementia and reflects imaging‐derived brain changes potentially related to neurodegeneration**.

